# Generation of Human Induced Pluripotent Stem Cells Using Epigenetic Regulators Reveals a Germ Cell-Like Identity in Partially Reprogrammed Colonies

**DOI:** 10.1371/journal.pone.0082838

**Published:** 2013-12-12

**Authors:** Akshi Goyal, Shawn L. Chavez, Renee A. Reijo Pera

**Affiliations:** 1 Center for Reproductive and Stem Cell Biology, Institute for Stem Cell Biology and Regenerative Medicine, Stanford, California, United States of America; 2 Department of Obstetrics and Gynecology, Stanford University School of Medicine, Stanford, California, United States of America; University of Muenster, Germany

## Abstract

Previous studies have shown that induced pluripotent stem cells (iPSCs) can be derived from fibroblasts by ectopic expression of four transcription factors, OCT4, SOX2, KLF4 and c-MYC using various methods. More recent studies have focused on identifying alternative approaches and factors that can be used to increase reprogramming efficiency of fibroblasts to pluripotency. Here, we use nucleofection, morpholino technologies and novel epigenetic factors, which were chosen based on their expression profile in human embryos, fibroblasts and undifferentiated/differentiated human embryonic stem cells (hESCs) and conventionally generated iPSCs, to reprogram human fibroblasts into iPSCs. By over expressing DNMT3B, AURKB, PRMT5 and/or silencing SETD7 in human fibroblasts with and without NANOG, hTERT and/or SV40 overexpression, we observed the formation of colonies resembling iPSCs that were positive for certain pluripotency markers, but exhibited minimal proliferation. More importantly, we also demonstrate that these partially-reprogrammed colonies express high levels of early to mid germ cell-specific genes regardless of the transfection approach, which suggests conversion to a germ cell-like identity is associated with early reprogramming. These findings may provide an additional means to evaluate human germ cell differentiation *in vitro*, particularly in the context of pluripotent stem cell-derived germ cell development, and contribute to our understanding of the epigenetic requirements of the reprogramming process.

## Introduction

Human embryonic stem cells (hESCs) are derived from the inner cell mass (ICM) of blastocysts and are characterized as pluripotent due to their ability to self-renew and give rise to all types of cells in the body. In 2007, Yamanaka and colleagues generated induced pluripotent stem cells (iPSCs), which are similar to hESCs in terms of morphology, gene expression and the ability to form cell types of all three germ layers both *in vitro* and *in vivo* [1-3]. iPSCs provide a platform for studying human development and disease as well as the potential to develop innovative patient-specific therapies with decreased risk of immune rejection relative to hECCs since the patient’s own cells might be used for therapy [4-7]. 

Initially, Yamanaka and colleagues reprogrammed fibroblasts by using four transcription factors (OCT4, SOX2, KLF4 and c-MYC) in viral vectors [3,8]. However, this method has several drawbacks and thus, recent studies have focused on eliminating the use of c-MYC and utilizing alternative methods of reprogramming, including excisable constructs, non-integrating plasmids adenovirus, episomal and *piggybac* transposon vectors to circumvent the genomic integration of viral transduction and increase reprogramming efficiency [9-10]. Other DNA-free methods such as Sendai virus, mRNA, microRNA and protein reprogramming have also been explored [11-15]. In general, two different approaches, the introduction of novel factors or the addition of cell permeable chemical compounds, either alone or in conjunction with one another have also been successfully used to increase the reprogramming efficiency of iPSCs. For the first approach, factors typically utilized for cell immortalization such as hTERT and the SV40 large T antigen have been transfected together with the four Yamanaka factors into human fetal, neonatal and adult dermal fibroblasts [16]. While hTERT and SV40 may increase cell growth in surviving colonies, they may also induce significant cell death and aneuploidy as previously shown [17]. Other studies have shown that additional novel factors, including NANOG, LIN28, REX1, Zfp296 and Glis1 can be used as a substitution together with one or more of the Yamanaka factors to make the reprogramming process more efficient [18]. Alternatively, iPSCs have also been derived using small molecule compounds such as Valproic Acid (VPA) and 5-Aza-2´-deoxycytidine (AZA), which may substitute for c-MYC during transfection and are thought to act by inducing epigenetic remodeling [19]. 

Despite recent studies demonstrating increased efficiency of iPSC generation, several reports have shown that iPSCs differ epigenetically from their hESC counterparts, which can be explained, at least in part, by incomplete DNA methylation or histone modification during cellular reprogramming [20-22]. Here, we explored use of various combinations of different novel epigenetic factors to reprogram human fibroblasts into iPSCs. We chose the factors used for reprogramming based on their expression profile in human embryos, fibroblasts and undifferentiated/differentiated hESCs and previously derived hiPSCs. By nucleofecting fibroblasts with plasmids containing DNMT3B, AURKB and PRMT5 in conjunction with SETD7 silencing via morpholino technologies, we demonstrate an early role for these epigenetic factors in reprogramming and reveal a germ cell-like identity in partially reprogrammed colonies. 

## Materials and Methods

### Ethics statement

Human blastocysts donated for non-stem research were obtained with written informed consent from the Stanford University Regenerative Medicine through the Ethical procurement of Nonviable or Excess cellular Waste (RENEW) Biobank as previously described [23]. De-identification and molecular analysis was performed according to the Stanford University Institutional Review Board (IRB)-approved protocol #10466 entitled “The RENEW Biobank.” No protected health information was associated with each of the blastocysts.

### Reagents and antibodies

The DNMT3B rabbit monoclonal antibody (clone #EPR3523) was purchased from Abcam (Cambridge, MA), whereas the SETD7 mouse monoclonal antibody (clone #5F2.3), Histone H3-K4me3 rabbit monoclonal antibody (clone #MC-315), SSEA3 rat monoclonal antibody (clone #MC-631) and TRA-1-60 mouse monoclonal (clone #TRA-1-60) were obtained from Millipore (Billerica, MA). While the AURKB goat polyclonal antibody (catalog #AF4006) was purchased from R&D Systems, Inc. (Minneapolis, MN), the PRMT5 rabbit monoclonal antibody (clone #EPR5772) was obtained from Epitomics (Burlingame, CA). The Histone H3-S28P rat monoclonal (clone HTA28) and the Histone H4-R3me2 rabbit polyclonal antibody (catalog #39275) were ordered from Thermo Fisher Scientific (Rockford, IL) and Active Motif (Carlsbad, CA), respectively. 2-propyl-pentanoic acid, monosodium salt (Valproic acid) was purchased from Millipore at used at a concentration of 1mM according to [19]. 5-aza-2′-deoxycytidine (AZA) was obtained from Sigma-Aldrich (Sigma-Aldrich, St. Louis, MO) and used at a 1μM concentration as previously described [24]. 

### Cell lines and culture conditions

The human adult dermal fibroblasts (AHDFs), known as HUF1 and HUF5, were obtained from a normal healthy male and female, respectively, as previously described [25]. HFF-1 (neonatal human foreskin fibroblasts) cells were purchased from ATCC (Manassas, VA; catalog #SCRC-1041). Human fibroblasts were cultured on gelatin-coated plates in DMEM high glucose Glutamax (Invitrogen; Carlsbad, CA) supplemented with 20% Fetal Bovine Serum (FBS; Invitrogen). The human embryonic stem cell lines, H9 (XX), HSF8 (XY) and HSF10 (XX) have been described previously [26] and were grown, along with the human neonatal and adult fibroblasts after nucleofection, on gelatin-coated plates containing gamma-irradiated mouse embryonic fibroblasts (MEFs) in hESC medium consisting of DMEM (Invitrogen), 20% knock-out serum replacer (Invitrogen), 10mM non-essential amino acids (Invitrogen), 200mM L-glutamine (Invitrogen) and 0.1mM β-mercaptoethanol (Invitrogen) and supplemented with 10ng/ml β-FGF (Peprotech; Rocky Hill, NJ). Both hESCs and hiPSCs were spontaneously differentiated via adherent culture for 7 to 21 days on matrigel without MEFs in DMEM (Invitrogen) supplemented with characterized HyClone 20% FBS (Thermo Fisher Scientific). For more directed differentiation to germ cells, hiPSCs were cultured adherently in differentiation media with 50ng/ml of recombinant human Bone Morphogenetic Protein 4 (BMP4), BMP7 and BMP8b (R&D Systems, Inc.) for 7 to 14 days as previously described [27,28]. Conditioned media (CM) was prepared by plating gamma-irradiated MEFs on gelatin-coated plates and culturing in hESC medium, which was collected everyday up to 5 days and filtered to remove residual MEFs before adding 10ng/ml β-FGF. The PGC media contained DMEM high glucose Glutamax supplemented with 20% FBS, 0.1μg/ml human recombinant Leukemia Inhibitory Factor (LIF) (Millipore) and 10ng/ml β-FGF. All cells were cultured at 37°C in 5% CO_2_.

### Plasmids, morpholinos and nucleofection

The DNMT3B plasmid (pCMV6-AC-GFP), AURKB plasmid (pCMV6-XL4) and PRMT5 plasmid (pCMV6-XL5) were purchased from Origene (Rockville, MD), whereas the Human TERT plasmid (pBABE-Neo), SV40 small + Large T antigen plasmid (pLenti-CMV-TO) and NANOG plasmid (EF1α-IRES-Puro) were obtained from Addgene (Cambridge, MA). The SETD7 morpholino with the sequence 5’- CCACCATCTCGTCGTCGCTATCCAT -3’, which was designed to target the translation start site of SETD7 and labeled with 3’-carboxyfluorescein for visualization, was ordered from Gene Tools, LLC (Philomath, OR). Human neonatal and adult fibroblasts were disaggregated into single cells using Accustase (Thermo Fisher Scientific) for nucleofection. 3μg of each plasmid and 30μM SETD7-MO were transfected using either the 96-well Amaxa Shuttle or 4D-Nucleofector X Unit from Lonza (Basel, Switzerland) and program #Y-023 with the NHDF Nucleofector Kit (Lonza) or program #EN-150 with the SE Cell Line 4D-Nucleofector X Kit (Lonza), respectively.

### Gene expression analysis

Pre-amplification was performed according to the manufacturer’s protocol (Fluidigm Corp.; So. San Francisco, CA) using the CellsDirect One-Step qRT-PCR kit (Invitrogen) and 20X TaqMan gene expression assays (Applied Biosystems, Foster City, CA). For undifferentiated and differentiated hESC lines and conventionally generated hiPSCs, RNA was first extracted using the Qiagen RNeasy Kit (Valencia, CA) and cDNA prepared with the SuperScript III First-Strand Synthesis System (Invitrogen). Between 1 and 250ng of cDNA was pre-amplified by adding 5μl CellsDirect 2X reaction mix, 2.5μl 0.2X PPP (2.5μl each 20X TaqMan gene expression assay plus Tris-EDTA buffer for a final volume of 250μl), 1μl Platinum Taq (Invitrogen), and 0.25-1.25μl TE buffer, thermocycled for 14 cycles at 95°C for 15 sec. and 60°C for 4 min. and the resulting PCR reactions diluted 1:2 with TE buffer. Nucleofected colonies were pre-amplified with 5μl CellsDirect 2X reaction mix, 2.5μl 0.2X PPP, 1μl SuperScript III/Platinum Taq (Invitrogen), 1.5μl TE buffer and 0.1μl Superase-In (Invitrogen), thermocycled at 50°C for 15 min., 70°C for 2 min, and for 18 cycles at 95°C for 15 sec. and 60°C for 4 min. before similarly diluting. 2.25μl of pre-amplified cDNA was mixed with 2.5μl of TaqMan Universal PCR Master Mix (Applied Biosystems) and 0.25μl of Fluidigm sample loading reagent. An assay mix was prepared by adding 2.5μl 20X TaqMan gene expression assay to 2.5μl Fluidigm assay loading reagent. Gene expression analysis was performed using 96.96 Dynamic Arrays (DA) and the BioMark microfluidic system (Fluidigm Corp.). Samples were analyzed in triplicate and their geometric mean calculated for normalization to the 2 or 3 most stable housekeeping genes using the qBasePlus 1.3 analysis software (http://www.biogazelle.com) as previously described [29] and graphed using Gene-E (http://www.broadinstitute.org/cancer/software/GENE-E/). 

### Human blastocyst thawing and culture

Human blastocysts were thawed using Quinn’s Advantage Thaw Kit (CooperSurgical, Trumbull, CT) as previously described [30]. In brief, cryostraws were removed from the liquid nitrogen and exposed to air before incubating in a 37°C water bath. Once thawed, the blastocysts were transferred to a 0.5-mol/L sucrose solution for 10 minutes followed by a 0.2-mol/L sucrose solution for an additional 10 minutes. The blastocysts were then washed in Quinn’s Advantage Medium with Hepes (CooperSurgical) plus 5% Serum Protein Substitute (SPS; CooperSurgical) and transferred to a 20μl microdrop of Quinn’s Advantage Blastocyst Medium (CooperSurgical) with 10% SPS under mineral oil (Sigma-Aldrich) for culturing at 37°C with 6% CO_2_, 5% O_2_ and 89% N_2_ for 2 hours prior to fixation. 

### Immunostaining

The zona pellucida (ZP) was removed from human blastocysts by treatment with Acidified Tyrode's Solution (Millipore) and ZP-free embryos were washed three times in Quinn’s Advantage Medium with Hepes plus 5% SPS before fixing in 4% paraforamaldehyde (USB Corp., Cleveland, OH) for 20 minutes. Undifferentiated and differentiated hiPSCs were plated on glass 4-well chamber slides (Lab-Tek; Hatfield, PA) and similarly fixed. Following fixation, the blastocysts, undifferentiated/differentiated hiPSCs and nucleofected clones were washed three times in Phosphate Buffered Solution (PBS; Invitrogen) with 0.1% Bovine Serum Albumin (BSA; Sigma-Aldrich) and 0.1% Tween-20 (PBS-T; Sigma-Aldrich). Permeabilization was performed by incubating in 0.1% Triton-X (Sigma-Aldrich) in PBS-T for 1 hour at room temperature (RT) and then washing three times in PBS-T. The blastocysts, undifferentiated/differentiated hiPSCs and nucleofected clones were blocked in 4% donkey serum (Jackson ImmunoReasearch Laboratories, Inc., West Grove, PA) in PBS-T at 4°C overnight and then incubated with primary antibodies for DNMT3B (1:100), SETD7 (1:500), AURKB (1:250) and PRMT5 (1:250), H3-S28P (1:200), H4-R3me2 (1:1,000), H3-K4me3 (1:200), SSEA3 (1:200) and/or TRA-1-60 (1:200) at RT for 1 hour. Primary signals were detected using the appropriate 488, 568 or 647-conjugated donkey Alexa Fluor secondary antibody (Invitrogen) at a 1:250 dilution at RT for 1 hour in the dark. The blastocysts, undifferentiated/differentiated hiPSCs and nucleofected clones were washed three times with PBS-T between each primary and secondary antibody incubation as well as prior to the next primary antibody incubation. Immunofluorescence was visualized by sequential imaging, whereby the channel track was switched each frame to avoid cross-contamination between channels, using a Zeiss LSM700 URGB Zen confocal microscope (Thornwood, NY). The instrument settings, including the laser power, pinhole and gain, were kept constant for each channel to facilitate semi-quantitiative comparisons between blastocysts, undifferentiated/differentiated hiPSCs and nucleofected clones.

## Results

### Gene expression profiling of epigenetic reprogramming factors in human fibroblasts, iPSCs and ESCs

**Figure 1:**
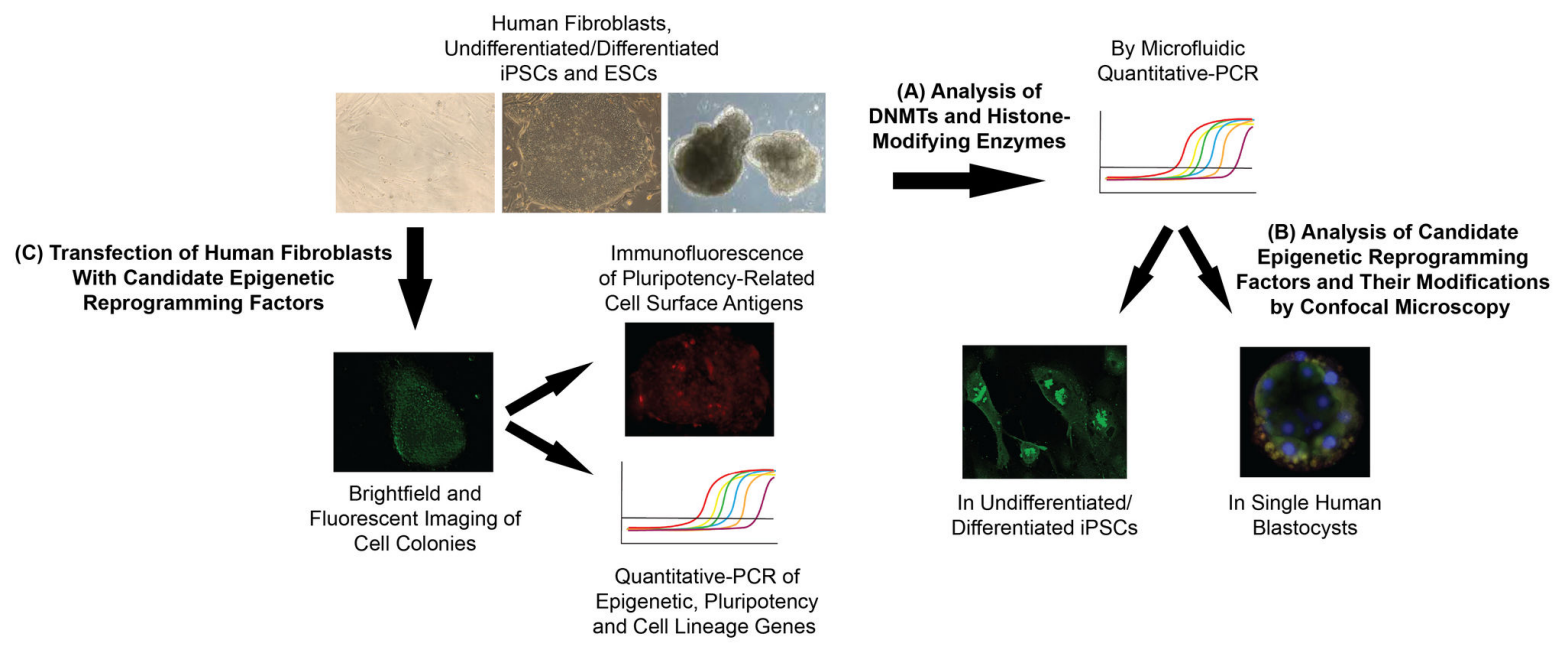
Experimental design of this study aimed at identifying novel epigenetic reprogramming factors. (**A**) We analyzed the expression of the DNA Methyltransferase (DNMT) family and members of the different histone-modifying enzyme classes in adult human fibroblasts, undifferentiated/differentiated human embryonic stem cells (hESCs) and human induced pluripotent stem cells (hiPSCs) by microfluidic Quantitative-PCR (Q-PCR) to identify candidate factors for epigenetic reprogramming. (**B**) The expression of the candidate reprogramming factors and the epigenetic modifications that they mediate were analyzed at the protein level in human blastocysts and undifferentiated/differentiated hiPSCs, respectively, by confocal microscopy. (**C**) Human neonatal and adult fibroblasts were transfected with the candidate epigenetic reprogramming factors, which were fluorescently-labeled for visualization, and colony formation assessed via brightfield and fluorescent imaging, immunofluorescence of pluripotency-related cell surface antigens and Q-PCR of epigenetic, pluripotency and cell lineage genes.

**Figure 2:**
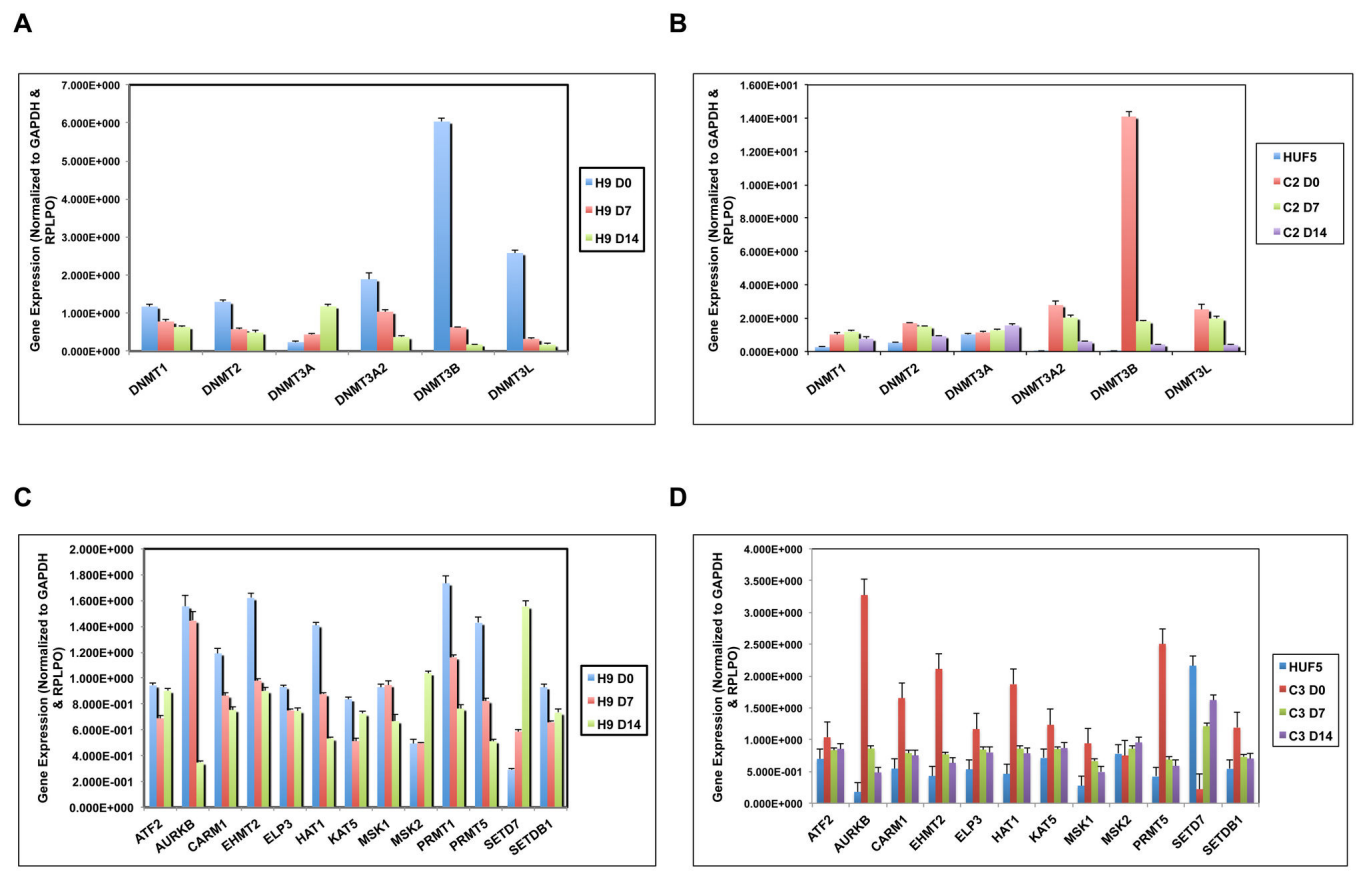
Gene expression analysis of candidate epigenetic reprogramming factors in hESCs and hiPSCs. The expression of DNA methyltransferases (DNMTs) was analyzed in undifferentiated (D0 for Day 0) as well as Day 7 (D7) and Day 14 (D14) differentiated (**A**) human embryonic stem cells (hESC; H9) and (**B**) human induced pluripotent stem cells (hiPSCs; clone 2) from normal adult human dermal fibroblasts (HUF5) by microfluidic Quantitative-PCR (Q-PCR). Cycle threshold (Ct) values were normalized to the two most stable housekeeping genes and graphed as shown. (**C**) Similar Q-PCR analysis of each histone-modifying enzyme class in H9 and (**D**) HUF5 clone 3. Note the high levels of DNMT3B, AURKB and *PRMT5* expression in undifferentiated hESCs and hiPSCs that decreases with differentiation and the elevated expression of SETD7 in D14 differentiated H9 cells and the original HUF5 fibroblasts to suggest that induction of DNMT3B, AURKB and PRMT5 expression in conjunction with SETD7 silencing may reprogram adult fibroblasts to a more hESC-like state.

We first evaluated the expression of both the DNA methyltransferase (DNMT) family and the different classes of histone-modifying enzymes at the mRNA level in three different hESC lines (H9, HSF8 and HSF10), two independently generated hiPSC lines (C2 and C3), and the original adult human dermal fibroblasts (AHDF) at day 0, 7, 14 and/or 21 of spontaneous differentiation by microfluidic Q-PCR (Figure **1A**). Our results suggest that the *de novo* methyltransferase, *DNMT3B*, is highly expressed in both undifferentiated hESCs and hiPSCs, whereas its expression is almost undetectable in non-reprogrammed AHDFs and decreases as hESC and hiPSC differentiation proceeds (Figure **2A, 2B** and Figure **S1**). Similarly, when we examined the expression of the different histone-modifying enzyme classes in AHDFs, hESCs, and the hiPSC clones we observed elevated expression of *AURKB* and *PRMT5*, enzymes that mediate the phosphorylation of serine residues and methylation of arginine residues, respectively, and their expression decreased upon differentiation (Figure **2C, 2D** and Figure **S2**). In contrast, high expression of the histone lysine methylation enzyme, *SETD7*, was detected in AHDFs, but not in undifferentiated hESCs and hiPSCS and then increased with differentiation between days 7, 14 and/or 21 (Figure **2C, 2D** and Figure **S2**). This suggested that the induction of DNMT3B, AURKB and PRMT5 expression in conjunction with the silencing of SETD7 expression might assist in the reprogramming of fibroblasts to a more hESC-like state.

### Analysis of candidate epigenetic reprogramming factors at the protein level in human blastocysts

**Figure 3:**
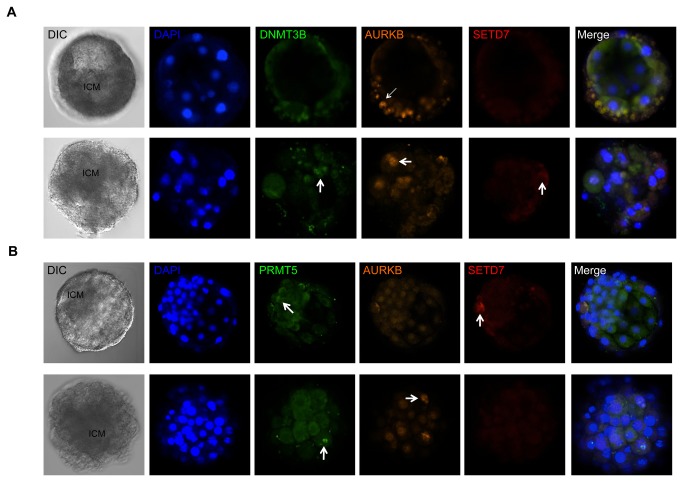
Expression and localization of candidate epigenetic reprogramming factors in human embryos. Human blastocysts were incubated with primary antibodies for (**A**) DNMT3B (green), AURKB (orange) and SETD7 (red) or (**B**) PRMT5 (green), AURKB (orange) and SETD7 (red), the nuclear DNA stained with DAPI (blue) and visualized by multi-channel confocal microscopy. Differential Interference Contrast (DIC) was used to distinguish the inner cell mass (ICM) from the trophoectoderm of each embryo. Note that the expression of DNMT3B, AURKB and PRMT5 primarily localized to the ICM of human blastocysts, whereas SETD7 expression was predominantly detected in the outer trophoectoderm layer of the embryos (indicated by white arrows).

Based on the data obtained with hESCs and hiPSCs here and our previous study of potential reprogramming candidates in human embryos and hESCs at the mRNA level (Chavez et al., unpublished data), we next examined the expression and localization of our candidate reprogramming factors at the protein level in human embryos (Figure **1B** and Figure **3**). For this purpose, human blastocysts were incubated with antibodies directed to DNMT3B, AURKB, PRMT5 and/or SETD7, stained with DAPI and analyzed by confocal fluorescent microscopy. As Figure 3A and 3B demonstrate, expression of DNMT3B, AURKB and PRMT5 was observed in human blastocysts and their expression primarily localized to the ICM of the embryo. In contrast, SETD7 expression was reduced in human blastocysts and was only detected within the trophoectoderm layer of the embryo (Figure 3A and 3B), confirming the mRNA expression profiling of these factors in human embryos, hESCs and iPSCs. 

### Assessment of histone modifications mediated by candidate epigenetic factors in hiPSCs

**Figure 4:**
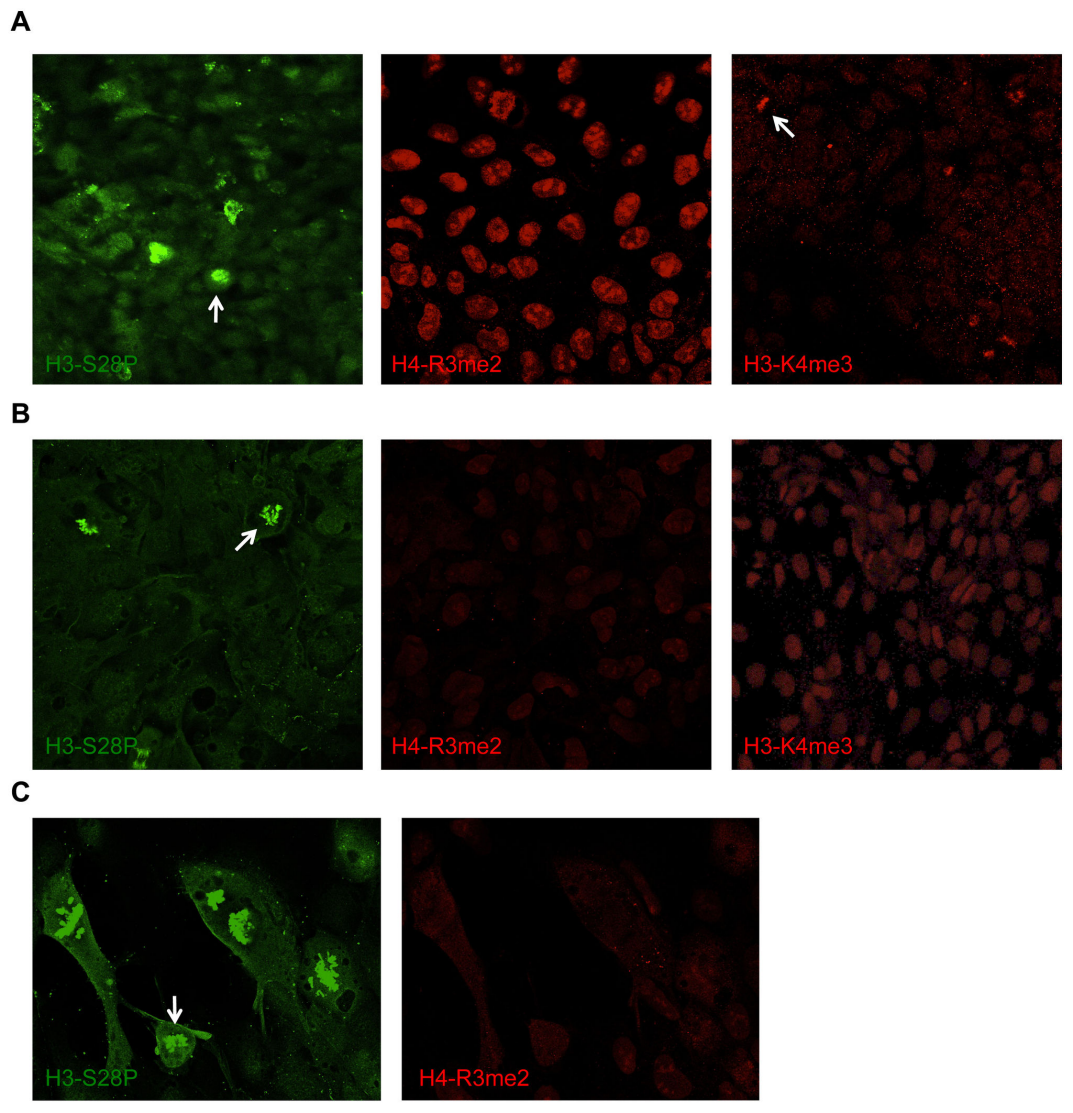
Confocal analysis of particular histone modifications in undifferentiated and differentiated hiPSCs. The expression of Histone H3-S28P (green), H4-R3me2 (red; middle panel) and H3-K4me3 (red; right panel), which are primarily mediated by AURKB, PRMT5 and SETD7, respectively, was evaluated in (**A**) undifferentiated and (**B**) Day 7 differentiated hiPSCs (HUF5 clone 2) by immunofluorescent confocal microscopy. Note that H3-K4me3 expression is increased or at least retained in hiPSCs upon differentiation. (**C**) Similar confocal analysis of histone modifications in Day 7 differentiated hiPSCs demonstrating that only a small population of cells still expresses H3-S28P, to suggest that they are proliferative, and decreased expression of H4-R3me2 in hiPSCs following 7 days of differentiation.

We next sought to further confirm our results at the protein level by immunostaining hESCs and hiPSCs with certain histone modifications that AURKB, PRMT5 and SETD7 are known to mediate (Figure **1B** and Figure **4**). While both Histone H3-serine 28 phosphorylation (H3-S28P) and Histone H4-arginine 3 dimethylation (H4-R3me2) were highly expressed in undifferentiated hiPSCs, their expression decreased upon differentiation (Figure 4A and 4B). However, there was a small population of differentiating hiPSCs that still expressed H3-S28P and since this epigenetic mark is associated with mitosis [31], this suggested that these cells are proliferative (Figure 4B and 4C; indicated by white arrows). Lower levels of Histone H3-lysine 4 tri-methylation (H3-K4me3), on the other hand, were detected in undifferentiated hiPSCs (indicated by white arrow) and its expression increased or was at least retained following differentiation in accordance with previous findings [32] to validate our findings at the mRNA level (Figure 4A and 4B).

### DNMT3B-GFP and SETD7-MO transfected colonies exhibit characteristics of partial reprogramming

**Figure 5:**
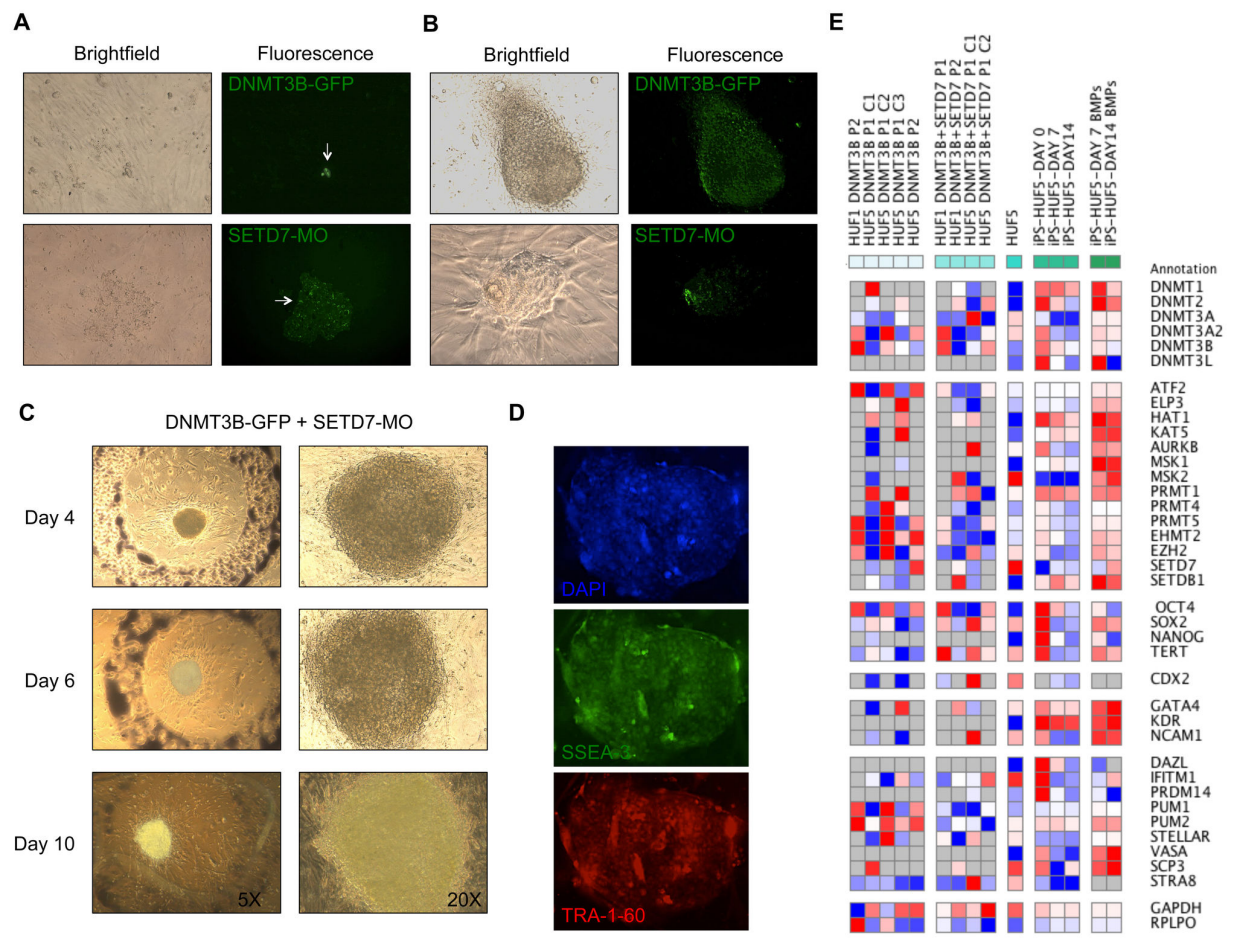
DNMT3B-GFP/SETD7-MO transfection induces the formation of colonies that are pluripotent, but exhibit minimal proliferation. Human adult dermal fibroblasts (HUF5) were nucleofected with a plasmid containing DNMT3B, which was tagged with GFP, and a 3’carboxyfluorescein (3’COF)-labeled SETD7 morpholino and the transfection efficiency assessed by epifluorescence. (**A**) The presence of DNMT3B-GFP and SETD7-MO was detected in the nucleus and cytoplasm, respectively, in transfected cells 2-3 days following nucleofection and (**B**) persisted in colonies that formed for several days afterwards. A greater number of clones appeared when the cells were transfected with both factors and the colonies obtained by nucleofection with DNMT3B-GFP appeared more hESC-like over those nucleofected with SETD7-MO alone. (**C**) When HUF5 cells were nucleofected with both DNMT3B-GFP and SETD-MO, the formation of several small clones was observed, which proliferated until approximately day 10. (**D**) Despite minimal proliferation, at least a portion of the colonies obtained by nucleofection with DNMT3B-GFP and SETD7-MO were positive for the pluripotency-related cell surface markers, Stage Specific Embryonic Antigen-3 (SSEA-3) and the keratin sulfate-related antigen, TRA-1-60. (**E**) Microfluidic Quantitative-PCR (Q-PCR) analysis of epigenetic, pluripotency and cell lineage genes in DNMT3B-GFP transfected HUF1 and HUF5 cells with and without SETD7-MO transfection compared to the original HUF5 adult fibroblasts and conventionally generated human induced pluripotent stem cells (hiPSCs) on day 0, 7 and 14 of differentiation with and without Bone Morphogenetic Proteins (BMPs). Note the similarities and differences in the expression of certain epigenetic regulators between DNMT3B-GFP and SETD7-MO nucleofected clones, undifferentiated and differentiated hiPSCs and adult fibroblasts as well as elevated expression of several pluripotency markers and germ cell-specific genes in DNMT3B-GFP/SETD7-MO transfected colonies. Grey squares indicate no expression, whereas blue, white and red squares correspond to low, medium and high expression, respectively. A direct comparison of global gene expression ratios between the different cell types is shown in Table **S2**.

Our results thus far suggested that the induction of DNMT3B, PRMT5 and AURKB expression and silencing of SETD7 expression in human fibroblasts might enhance the reprogramming of fibroblasts into iPSCs. To test this theory, we first nucleofected adult human fibroblasts (HUF1 and HUF5) with a plasmid encoding DNMT3B and/or a SETD7 morpholino (SETD7-MO; Figure **1C**) given that morpholinos can be easily transfected into cells using nucleofection technologies [33]. For visualization and to assess transfection efficiency, the DNMT3B overexpression vector was GFP-tagged and the SETD7 morpholino 3’-carboxyfluroescein (3’-COF) labeled. As shown in Figure **5A**, the presence of GFP in the nucleus and 3’COF in the cytoplasm was detected in some cells transfected with the DNMT3B plasmid or SETD7-MO 2-3 days post-transfection, respectively, indicating that nucleofection was sufficient for introducing these factors into fibroblasts. However, fewer colonies appeared when the cells were transfected with only one factor and the colonies obtained by nucleofection with DNMT3B-GFP appeared more hESC-like over those nucleofected with the SETD7-MO alone (Figure **5B**). When fibroblasts were nucleofected with both DNMT3B-GFP and SETD-MO, the formation of several small clones was observed, which proliferated until day 10 (Figure **5C**) and then underwent senescence after approximately 14 days post-nucleofection (Figure **S3A**; left). Therefore, we performed a second round of nuclefoection with DNMT3B-GFP and SETD7-MO between 2 to 7 days as previously described [34] and determined that nucloefecting cells on Day 1 and 3 was the most efficient in terms of reprogramming efficiency and number of clones (Table **S1** and Figure **S3B**). Nucleofecting fibroblasts with DNMT3B-GFP and SETD7-MO more than two times, however, did not yield better results due to cellular detachment for nucleofection and massive cell death even in the presence of conditioned media on matrigel or mouse embryonic fibroblasts (MEFs) used as feeders (Figure **S3A**; right). Despite minimal proliferation, at least some of the cells in the colonies obtained by nucleofection with DNMT3B-GFP and SETD7-MO were positive for pluripotency markers such as Stage Specific Embryonic Antigen-3 (SSEA-3) and the keratin sulfate-related antigen, TRA-1-60 (Figure **5D**).

### Gene expression analysis of DNMT3B-GFP and SETD7-MO transfected clones

Our next objective was to perform gene expression analysis of the clones we obtained by nucleofecting fibroblasts with DNMT3B-GFP and/or SETD7-MO by microfluidic Q-PCR (Figure **1C**). For comparison, we also included undifferentiated iPSCs derived by similarly introducing the four Yamanaka factors by nucleofection into the adult human fibroblasts and spontaneously differentiated on day 7 and 14 in the analysis. As Figure **5E** demonstrates, several clones expressed high levels of *DNMT3B* and this expression occasionally persisted even after passaging. The expression of other DNMTs such as *DNMT3A2* was also observed in some of the clones and correlated with the induction of *DNMT3B*, however, *DNMT3L* expression was not detected in any of the clones (Figure **5E**). Unlike *DNMT3B*, the expression of *SETD7* was low or absent in SETD7-MO-nucleofected clones, but its expression returned upon passaging (Table **S2**). Analogous to DNMT3B alone, however, the combination of DNMT3B over expression and SETD7 silencing had effects on the expression of other epigenetic modifiers in colonies derived from adult human fibroblasts (Figure **5E**). 

In support of the SSEA-3 and TRA-1-60 partial immunostaining data (Figure **5D**), we observed expression of the major pluripotency expressed gene, *OCT4*, at levels comparable to undifferentiated iPSCs generated with the four Yamanaka factors in several of the DNMT3B-GFP and/or SETD7-MO transfected clones (Figure **5E**). However, while certain clones also expressed additional pluripotency markers such as *SOX2* and *hTERT*, *NANOG* was not expressed in the majority of clones (Table **S2**), suggesting that introduction of DNMT3B-GFP and/or SETD7-MO induced partially reprogramming of adult human fibroblasts. An examination of the trophoblast marker, *CDX2*, and the three germ layer genes, *GATA4*, *KDR* and *NCAM1* did not indicate that the colonies had formed other obvious cell types representative of extraembryonic or germ layer tissues (Figure **5E**). Notably, when we analyzed the expression of germ cell-specific genes, however, we observed high levels of early to mid germ cell markers, including Pumilio Homolog 1 (*PUM1*), *PUM2* and *STELLAR* (*DPPA3*) in the clones (Figure **5E** and Table **S2**). Plating the nucleofected cells on STO feeders and culturing the colonies in Primordial Germ Cell (PGC) media containing LIF and FGF, however, did not change their morphology (data not shown). Nevertheless, a comparison of gene expression between the clones and hiPSCs differentiated in the presence of BMPs, demonstrated similar expression levels of early and mid germ cell-specific genes, but also high expression of late germ cell markers such as *VASA* (*Mvh*) and Synaptonemal Complex Protein 3 (SCP3) only in the BMP-differentiated hiPSCs. Since the addition of BMPs is generally used for more directed germ cell differentiation of pluripotent stem cell cultures, this suggested that the partially-reprogrammed clones exhibited a gene expression profile reminiscent of early stage hiPSC-derived germ cells. 

### Addition of AURKB, PRMT5, VPA and/or AZA for increasing reprogramming efficiency

**Figure 6:**
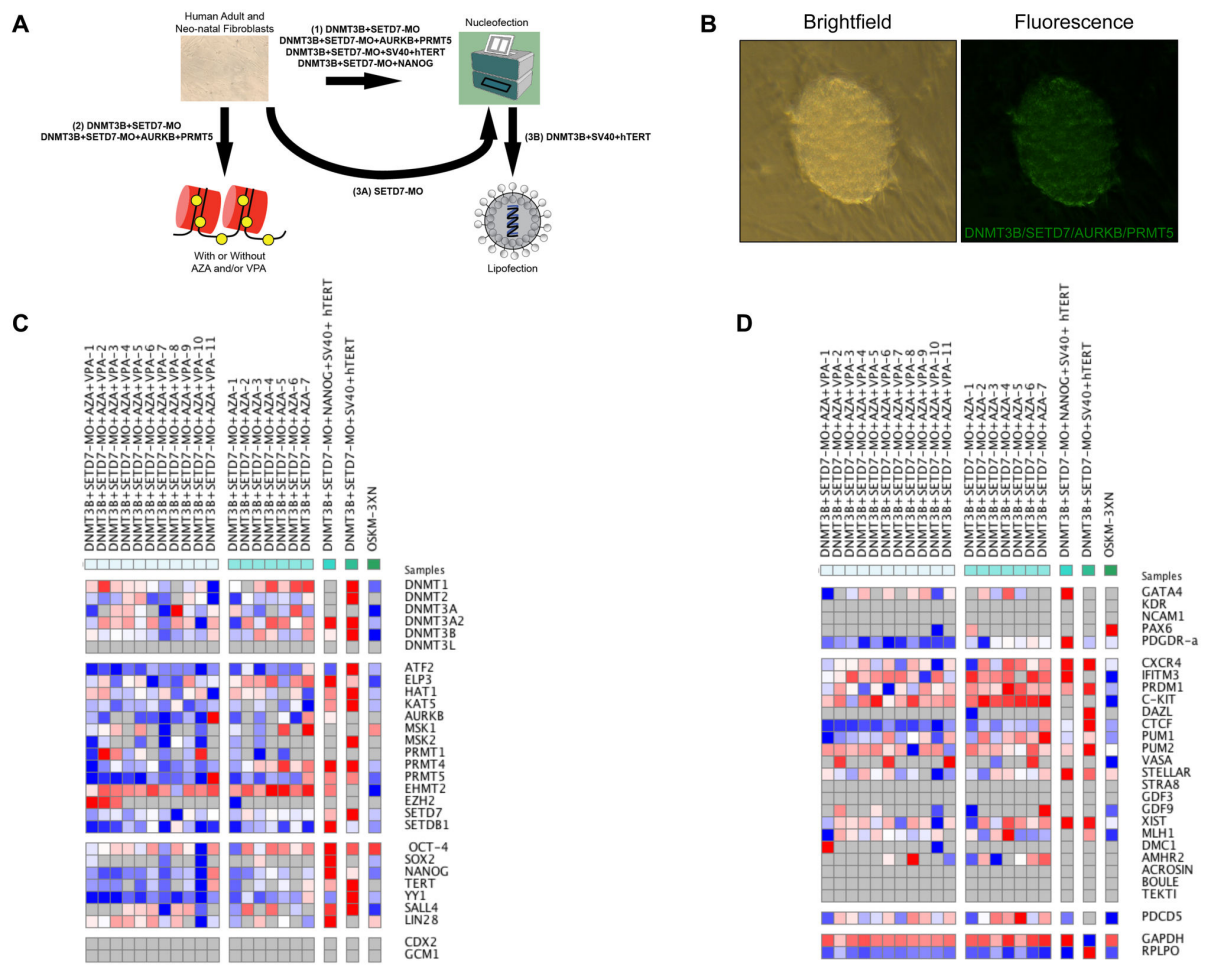
Addition of other factors and chemical compounds for increasing epigenetic reprogramming efficiency. (**A**) The experimental design for increasing reprogramming efficiency in human adult, neonatal and fetal fibroblasts with the use of (1) the additional reprogramming factors, AURKB, PRMT5, SV40, hTERT and/or NANOG, (2) the cell-permeable chemical compounds, AZA and/or VPA, to assist in chromatin remodeling for gene activation and (3) an alternative transfection approach whereby fibroblasts are first nucleofected with SETD7-MO and then transfected with DNMT3B, hTERT and SV40 via a cationic lipid reagent. (**B**) Brightfield imaging of colonies obtained by nucleofecting neonatal human foreskin fibroblasts (HFF-1) with DNMT3B-GFP/SETD7-MO in the presence of AZA and VPA, which represented the largest number of clones obtained. (**C**) Gene expression analysis of epigenetic regulators, pluripotency genes and trophoectoderm markers as well as (**D**) markers of the three germ layers and germ-cell specific genes in HFF-1 cells transfected with different combinations of reprogramming factors and chemicals by microfluidic Quantitative-PCR (Q-PCR) for comparison to fibroblasts similarly nucleofected three times with the Yamanaka factors (OCT4, SOX2, KLF4 and c-MYC). Note the lack or negligible expression of trophoectoderm and germ layer markers, but high levels of early and mid germ cell-specific genes in HFF-1 colonies not observed in conventionally generated hiSPCs. Grey squares indicate no expression, whereas blue, white and red squares correspond to low, medium and high expression, respectively.

Based on our findings that the colonies we obtained by DNMT3B-GFP and SETD7-MO transfection appeared to be only partially reprogrammed and exhibited characteristics of germ cells, we next sought to include additional candidate epigenetic factors and particular chemical compounds for increasing the reprogramming efficiency (Figure **6A**). To accomplish this, we used the neonatal human foreskin fibroblast line, HFF-1, as well as adult human fibroblasts since fibroblasts isolated from earlier developmental stages can be more easily reprogrammed [35]. In addition, we nucleofected HFF-1 and HUF5 fibroblasts with different combinations of DNMT3B-GFP, SETD7-MO as well as expression vectors for AURKB and PRMT5 (Figure **6B** and Figure **S3C**), the other histone-modifying enzymes also shown to be highly expressed in undifferentiated hESCs and hiPSCs that decreased upon differentiation (Figure **2C, 2D** and Figure **S2**). Finally, the HFF-1 cells were pre-treated with AZA for three days prior to transfection, nucleofected and then incubated with or without VPA (Figure **6A**) based on the assumption that these cell permeable compounds help to open up the chromatin for gene activation [36]. While pre-incubation of the neonatal fibroblasts with AZA induced cell death (Figure **S4A**), the addition of VPA to the culture media significantly increased colony formation (Table **S1** and Figure **S4B**). Moreover, the induction of AURKB and PRMT5 expression together with DNMT3B over expression and SETD7 silencing also resulted in reduced colony formation and cell death, which may be due to the large amount of DNA needed to transfect the HUF1 and HFF-1 fibroblasts with all four factors and (Figure **6B**, Table **S1** and Figure **S3C**). Analogous to the results observed with the adult human fibroblasts, some of the largest number of clones was obtained when the human neonatal fibroblasts were nucleofected with both DNMT3B-GFP and SETD7-MO in the absence or presence of AZA and/or VPA (Table **S1** and Figure **S4A** and **S4B**). 

Analysis of gene expression in the resulting clones from HFF-1 fibroblasts revealed similar effects of DNMT3B-GFP and SETD7-MO nucleofection on the expression of DNMTs and histone-modifying enzymes, including the lack of *DNMT3L* expression (Figure **6C**) as in adult dermal fibroblasts. In addition, the majority of colonies also expressed relatively high levels of *OCT4* as well as other pluripotency markers such as *SALL4* and *LIN28* to suggest that the cells were at least partially pluripotent. Unlike the clones observed with adult fibroblasts (Figure **5E**), however, *NANOG* was expressed in almost all of the colonies obtained from neonatal fibroblasts (Figure **6C**). Moreover, while trophoblast genes such as *CDX2* and *GCM1* were not expressed in any of the clones, markers of the three germ layers, including *GATA4*, *KDR*, *NCAM1*, *PAX6* and *PDGDR-α*, were either not expressed or expressed at relatively low levels (Figure **6D**). Notably, evaluation of the same germ cell genes in neonatal and adult fibroblasts or additional germ cell markers in HFF-1 cells suggested that the colonies appeared to be germ cell-like, unlike conventionally generated colonies (Figure **5E** and Figure **6D**). In particular, early germ cell-specific genes such as Chemokine (C-X-C Motif) Receptor 4 (*CXCR4*), *IFITM3* (*Fragilis*), PR domain zinc finger protein 1 (*PRDM1*;*BLIMP1*), *c-KIT* as well as *PUM1*, *PUM2* and *STELLAR* were highly expressed in several HFF-1 clones. Analogous to the BMP differentiated hiPSCs shown in Figure **5E**, some of the HFF-1 colonies also expressed high levels of *VASA* to suggest that the neonatatal cells are indeed more easily reprogrammable (Figure **6D**). Later germ cell, meiosis-related or sex-specific markers, including *STRA8*, *GDF3*, *ACROSIN*, *DMC1*, *ACROSIN*, *BOULE* and *TEKT1*, on the other hand, were not expressed or exhibited negligible expression in neonatal colonies (Figure **6D**). 

### Increasing reprogramming efficiency by the addition of NANOG and/or SV40 and hTERT

Previous studies have shown that NANOG is one of the most important pluripotency factors and an indication of complete reprogramming to pluripotency [37]. Given that we did not observe high levels of *NANOG* expression in the majority of clones we obtained with DNMT3B-GFP and SETD7-MO, we included NANOG with SV40 and hTERT, which have shown to increase reprogramming efficiency due to their immortalization properties [16,17], to the reprogramming factor cocktail (Figure **6A**). After 5 to 7 days post-nucleofection, several small clones formed (Table **S1** and Figure **S4C** and **S4D**) and expressed high levels of almost all pluripotency genes tested, but did not proliferate (Figure **6D**). Nucleofecting neonatal fibroblasts more than two times did not assist in generating fully-reprogrammed clones as significant cell death occurred upon cellular detachment. In order to circumvent this, the HFF-1 cells were nucleofected with SETD7-MO and then transfected with DNMT3B, SV40 and hTERT using a cationic lipid reagent (Figure **6A** and Table **S1**). Although the numerous clones we observed early in the reprogramming process by this method did not express pro-apoptotic genes such as PDCD5, after approximately two weeks, these colonies began to undergo apoptosis (data not shown). Nevertheless, early germ cell markers were highly expressed in colonies obtained by the addition of NANOG, SV40 and/or hTERT, suggesting that the partially-reprogrammed clones exhibit a germ cell-like identity regardless of the type of transfection method used (Figure **6D**). In addition, including NANOG in the reprogramming cocktail with or without SV40 and hTERT also significantly changed the morphology of the colonies to a more embryonic germ (EG) cell-like appearance, namely a more rounded shape and less flattened edges (Figure **S4C** and **S4D**).

## Discussion

Since the advent of hiPSCs a few years ago [3,8], several studies have focused on identifying and utilizing novel factors, cell-permeable compounds and/or different transfection approaches for increasing the efficiency of reprogramming human fibroblasts to pluripotency [38]. Here, we overexpressed the epigenetic factors, *DNMT3B*, *AURKB* and *PRMT5* and silenced *SETD7* expression based on their expression profile in human embryos, fibroblasts and undifferentiated/differentiated hESCs and conventionally generated hiPSCs, to reprogram neonatal and adult fibroblasts into hiPSCs. In conjunction with DNMT3B, AURKB and PRMT5 overexpression and SETD7 silencing, we also treated fibroblasts with the small molecule compounds, VPA and AZA, and included additional factors such as NANOG, SV40 and/or hTERT for reprogramming. From these experiments, we did not obtain fully-reprogrammed clones using these different factors and various methods, however, we observed that cells acquired a germ cell-like identity that was associated with early reprogramming.

After determining which DNMTs and histone-modifying enzymes to overexpress or silence, we transfected fibroblasts by nucleofection since morpholinos can be easily introduced into cells by this method [33] and to avoid the genomic integration associated with viral technologies [9]. Our data demonstrates that the introduction of two factors, DNMT3B-GFP and SETD7-MO, in both neonatal and adult fibroblasts was the most efficient in terms of colony number and proliferation. The decreased colony formation observed with the induction of *AURKB*, *PRMT5*, *NANOG*, *SV40* and/or *hTERT* expression may be due to the excess amount of DNA required for transfection or the association of some of these factors with cell death [17]. Moreover, the number of clones obtained by first treating fibroblasts with AZA for three days prior to nucleofection in media containing VPA was much greater than with AZA pre-treatment alone, which may be explained by previous findings that AZA can induce apoptosis in mammalian cells [39].

Regardless of the approach, the colonies that formed following transfection appeared pluripotent based on expression of pluripotency-related cell surface antigens and pluripotency genes at the protein and mRNA level, respectively. In addition, while certain clones exhibited cell proliferation early in the reprogramming process, this proliferation ceased after two weeks and the cells began to undergo apoptosis. This may help explain why only certain cells, rather than all or at least the majority of cells as in typical hESC and iPSC colonies, were positive for pluripotency-related cell surface antigens. More importantly, we observed high levels of several early to mid germ cell markers in colonies obtained by different reprogramming methods from both neonatal and adult fibroblasts that were collected prior to this two-week period. Notably, the germ cell-specific gene, *PRDM1/BLIMP-1*, was previously shown to be a target of *LIN28*, one of the four factors that Thomson and colleagues originally introduced for reprogramming somatic cells to pluripotency[40], via inhibition of the let-7 microRNA[41]. Although LIN28 is not essential for reprogramming process, it does increase reprogramming efficiency[40], and given recent findings of inverse correlation between let-*7* and *Prdm1* expression in mouse iPSCs[42], suggests that the induction of germ cell-specific genes is part of the reprogramming process. Based on the assumption that partially-reprogrammed iPSCs represent intermediate stages of reprogramming[43,44] and given that naturally developing germ cells are also pluripotent and proliferate only at distinct times during development [45], perhaps a germ cell-like state is associated with the transition of somatic cells to reprogrammed iPSCs as previously described [42,46,47]. In support of this, we observed high to moderate levels of *LIN28* and *PRDM1* expression in almost all of the clones we obtained from the neonatal fibroblasts. Finally, the addition of NANOG, which has been show to be dispensible for somatic pluripotency, but required for germ cell formation [48], to the reprogramming cocktail changed the colony morphology to a more EG cell-like appearance. Thus, our findings may provide an additional means to evaluate human germ cell differentiation *in vitro*, particularly in the context of pluripotent stem cell-derived germ cell development, and assist in our understanding of complete epigenetic reprogramming. Additional research should be aimed at characterizing the cell fate of the germ-like cells *in vitro* and *in vivo* using a xeno-transplant model. 

## Supporting Information

Figure S1 **Analysis of DNMT family member expression in additional hESC and hiPSC lines.** The expression of each member of the DNA methyltransferase (DNMT) family was analyzed in undifferentiated (D0 for Day 0) as well as Day 7 (D7), Day 14 (D14) and Day 21 (D21) differentiated human embryonic stem cells (hESCs) lines, HSF8 and HSF10, the original adult dermal fibroblasts (HUF5), undifferentiated (D0) human induced pluripotent stem cells (hiPSCs; clone 3), D7 and D14 differentiated hiPSCs by microfluidic Quantitative-PCR (Q-PCR).
(DOCX)

Figure S2 **Quantification of histone-modifying enzyme expression in other hESCs and hiPSC lines.** The expression of members from each class of histone-modifying enzymes was analyzed in undifferentiated (D0 for Day 0) as well as Day 7 (D7), Day 14 (D14) and/or Day 21 (D21) differentiated human embryonic stem cells (hESCs), HSF8 and HSF10, the original adult dermal fibroblasts (HUF5), undifferentiated (D0) human induced pluripotent stem cells (hiPSCs; clone 2), D7 and D14 differentiated hiPSCs by microfluidic Quantitative-PCR (Q-PCR).
(DOCX)

Figure S3 **Brightfield and fluorescent imaging of additional colonies obtained from human adult fibroblasts.** Human adult dermal fibroblasts (HUF1 or HUF5) were nucleofected with DNMT3B-GFP and SETD7-MO or DNMT3B-GFP, SETD7-MO, AURKB and PRMT5 and colony formation assessed via brightfield and fluorescent imaging.
(DOCX)

Figure S4 **Additional colonies obtained from various reprogramming strategies using human neonatal fibroblasts.** Neonatal human foreskin fibroblasts (HFF-1) were treated with 5-Aza-2´-deoxycytidine (AZA) and/or Valproic Acid (VPA) in combination with DNMT3B-GFP, SETD7-MO and NANOG or DNMT3B-GFP, SETD7-MO, NANOG, SV40 and hTERT nucleofection and colony formation assessed via brightfield imaging.
(DOCX)

Table S1 **The reprogramming efficiency of each transfection approach.** A table displaying the cell type, transfection method, reprogramming factors and treatment conditions used for each transfection approach in this study.
(DOCX)

Table S2 **Comparison of gene expression ratios between cell types.** A table comparing global gene expression levels of pluripotency factors, the candidate reprogramming factors (DNMT3B and/or SETD7) and germ cell markers in the original HUF5 adult dermal human fibroblasts, following transfection with DNMT3B and/or SETD7-MO with passage (P) and clone (C) numbers, and conventionally generated induced pluripotent stem cells (iPS) on Day 0, 7 and 14 of differentiation with and without Bone Morphogenetic Proteins (BMPs).
(DOCX)

## References

[1] Okita K, Nakagawa M, Hyenjong H, Ichisaka T, Yamanaka S (2008) Generation of mouse induced pluripotent stem cells without viral vectors. Science 322: 949-953. doi:10.1126/science.1164270. PubMed: 18845712. 18845712

[2] Nakagawa M, Koyanagi M, Tanabe K, Takahashi K, Ichisaka T et al. (2008) Generation of induced pluripotent stem cells without Myc from mouse and human fibroblasts. Nat Biotechnol 26: 101-106. doi:10.1038/nbt1374. PubMed: 18059259. 18059259

[3] Takahashi K, Tanabe K, Ohnuki M, Narita M, Ichisaka T et al. (2007) Induction of pluripotent stem cells from adult human fibroblasts by defined factors. Cell 131: 861-872. doi:10.1016/j.cell.2007.11.019. PubMed: 18035408. 18035408

[4] Meyer JR (2008) The significance of induced pluripotent stem cells for basic research and clinical therapy. J Med Ethics 34: 849-851. doi:10.1136/jme.2008.024786. PubMed: 19043107. 19043107

[5] Ye L, Swingen C, Zhang J (2013) Induced pluripotent stem cells and their potential for basic and clinical sciences. Curr Cardiol Rev 9: 63-72. doi:10.2174/157340313805076278. PubMed: 22935022. 22935022 PMC3584308

[6] Rashid ST, Alexander GJ (2013) Induced pluripotent stem cells: From Nobel Prizes to clinical applications. J Hepatol 58: 625-629. doi:10.1016/j.jhep.2012.10.026. PubMed: 23131523. 23131523

[7] Ferreira LM, Mostajo-Radji MA (2013) How induced pluripotent stem cells are redefining personalized medicine. Gene 520(1): 1-6. doi:10.1016/j.gene.2013.02.037. PubMed: 23470844. 23470844

[8] Okita K, Ichisaka T, Yamanaka S (2007) Generation of germline-competent induced pluripotent stem cells. Nature 448: 313-317. doi:10.1038/nature05934. PubMed: 17554338. 17554338

[9] Yu J, Hu K, Smuga-Otto K, Tian S, Stewart R et al. (2009) Human induced pluripotent stem cells free of vector and transgene sequences. Science 324: 797-801. doi:10.1126/science.1172482. PubMed: 19325077. 19325077 PMC2758053

[10] Woltjen K, Michael IP, Mohseni P, Desai R, Mileikovsky M et al. (2009) piggyBac transposition reprograms fibroblasts to induced pluripotent stem cells. Nature 458: 766-770. doi:10.1038/nature07863. PubMed: 19252478. 19252478 PMC3758996

[11] Fusaki N, Ban H, Nishiyama A, Saeki K, Hasegawa M (2009) Efficient induction of transgene-free human pluripotent stem cells using a vector based on Sendai virus, an RNA virus that does not integrate into the host genome. Proc Jpn Acad Ser B Phys Biol Sci 85 pp. 348-362. PubMed: 19838014. 10.2183/pjab.85.348 PMC3621571 19838014

[12] Warren L, Manos PD, Ahfeldt T, Loh YH, Li H et al. (2010) Highly efficient reprogramming to pluripotency and directed differentiation of human cells with synthetic modified mRNA. Cell Stem Cell 7: 618-630. doi:10.1016/j.stem.2010.08.012. PubMed: 20888316. 20888316 PMC3656821

[13] Miyoshi N, Ishii H, Nagano H, Haraguchi N, Dewi DL et al. (2011) Reprogramming of mouse and human cells to pluripotency using mature microRNAs. Cell Stem Cell 8: 633-638. doi:10.1016/j.stem.2011.05.001. PubMed: 21620789. 21620789

[14] Kim D, Kim CH, Moon JI, Chung YG, Chang MY et al. (2009) Generation of human induced pluripotent stem cells by direct delivery of reprogramming proteins. Cell Stem Cell 4: 472-476. doi:10.1016/j.stem.2009.05.005. PubMed: 19481515. 19481515 PMC2705327

[15] Zhou H, Wu S, Joo JY, Zhu S, Han DW et al. (2009) Generation of induced pluripotent stem cells using recombinant proteins. Cell Stem Cell 4: 381-384. doi:10.1016/j.stem.2009.04.005. PubMed: 19398399. 19398399 PMC10182564

[16] Mali P, Ye Z, Hommond HH, Yu X, Lin J et al. (2008) Improved efficiency and pace of generating induced pluripotent stem cells from human adult and fetal fibroblasts. Stem Cells 26: 1998-2005. doi:10.1634/stemcells.2008-0346. PubMed: 18511599. 18511599

[17] Park IH, Zhao R, West JA, Yabuuchi A, Huo H et al. (2008) Reprogramming of human somatic cells to pluripotency with defined factors. Nature 451: 141-146. doi:10.1038/nature06534. PubMed: 18157115. 18157115

[18] Maekawa M, Yamaguchi K, Nakamura T, Shibukawa R, Kodanaka I et al. (2011) Direct reprogramming of somatic cells is promoted by maternal transcription factor Glis1. Nature 474: 225-229. doi:10.1038/nature10106. PubMed: 21654807. 21654807

[19] Huangfu D, Osafune K, Maehr R, Guo W, Eijkelenboom A et al. (2008) Induction of pluripotent stem cells from primary human fibroblasts with only Oct4 and Sox2. Nat Biotechnol 26: 1269-1275. doi:10.1038/nbt.1502. PubMed: 18849973. 18849973

[20] Kim K, Doi A, Wen B, Ng K, Zhao R et al. (2010) Epigenetic memory in induced pluripotent stem cells. Nature 467: 285-290. doi:10.1038/nature09342. PubMed: 20644535. 20644535 PMC3150836

[21] Lister R, Pelizzola M, Kida YS, Hawkins RD, Nery JR et al. (2011) Hotspots of aberrant epigenomic reprogramming in human induced pluripotent stem cells. Nature 471: 68-73. doi:10.1038/nature09798. PubMed: 21289626. 21289626 PMC3100360

[22] Nazor KL, Altun G, Lynch C, Tran H, Harness JV et al. (2012) Recurrent variations in DNA methylation in human pluripotent stem cells and their differentiated derivatives. Cell Stem Cell 10: 620-634. doi:10.1016/j.stem.2012.02.013. PubMed: 22560082. 22560082 PMC3348513

[23] Kalista T, Freeman HA, Behr B, Pera RR, Scott CT (2011) Donation of embryos for human development and stem cell research. Cell Stem Cell 8: 360-362. doi:10.1016/j.stem.2011.02.018. PubMed: 21474099. 21474099

[24] Han J, Sachdev PS, Sidhu KS (2010) A combined epigenetic and non-genetic approach for reprogramming human somatic cells. PLOS ONE 5: e12297. doi:10.1371/journal.pone.0012297. PubMed: 20808872. 20808872 PMC2924394

[25] Byrne JA, Nguyen HN, Reijo Pera RA (2009) Enhanced generation of induced pluripotent stem cells from a subpopulation of human fibroblasts. PLOS ONE 4: e7118. doi:10.1371/journal.pone.0007118. PubMed: 19774082. 19774082 PMC2744017

[26] Chavez SL, Meneses JJ, Nguyen HN, Kim SK, Pera RA (2008) Characterization of six new human embryonic stem cell lines (HSF7, -8, -9, -10, -12, and -13) derived under minimal-animal component conditions. Stem Cells Dev 17: 535-546. doi:10.1089/scd.2007.0216. PubMed: 18513167. 18513167

[27] Kee K, Gonsalves JM, Clark AT, Pera RA (2006) Bone morphogenetic proteins induce germ cell differentiation from human embryonic stem cells. Stem Cells Dev 15: 831-837. doi:10.1089/scd.2006.15.831. PubMed: 17253946. 17253946

[28] Kee K, Angeles VT, Flores M, Nguyen HN, Reijo Pera RA (2009) Human DAZL, DAZ and BOULE genes modulate primordial germ-cell and haploid gamete formation. Nature 462: 222-225. doi:10.1038/nature08562. PubMed: 19865085. 19865085 PMC3133736

[29] Hellemans J, Mortier G, De Paepe A, Speleman F, Vandesompele J (2007) qBase relative quantification framework and software for management and automated analysis of real-time quantitative PCR data. Genome Biol 8: R19. doi:10.1186/gb-2007-8-2-r19. PubMed: 17291332. 17291332 PMC1852402

[30] Chavez SL, Loewke KE, Han J, Moussavi F, Colls P et al. (2012) Dynamic blastomere behaviour reflects human embryo ploidy by the four-cell stage. Nat Comm. p. 1251. p. 10.1038/ncomms2249 PMC3535341 23212380

[31] Goto H, Yasui Y, Nigg EA, Inagaki M (2002) Aurora-B phosphorylates Histone H3 at serine28 with regard to the mitotic chromosome condensation. Genes Cells 7: 11-17. doi:10.1046/j.1356-9597.2001.00498.x. PubMed: 11856369. 11856369

[32] Agger K, Cloos PA, Christensen J, Pasini D, Rose S et al. (2007) UTX and JMJD3 are histone H3K27 demethylases involved in HOX gene regulation and development. Nature 449: 731-734. doi:10.1038/nature06145. PubMed: 17713478. 17713478

[33] Owen LA, Uehara H, Cahoon J, Huang W, Simonis J et al. (2012) Morpholino-mediated increase in soluble Flt-1 expression results in decreased ocular and tumor neovascularization. PLOS ONE 7: e33576. doi:10.1371/journal.pone.0033576. PubMed: 22438952. 22438952 PMC3305322

[34] Si-Tayeb K, Noto FK, Sepac A, Sedlic F, Bosnjak ZJ et al. (2010) Generation of human induced pluripotent stem cells by simple transient transfection of plasmid DNA encoding reprogramming factors. BMC Dev Biol 10: 81. doi:10.1186/1471-213X-10-81. PubMed: 20682060. 20682060 PMC2923111

[35] Yamaguchi T, Hamanaka S, Kamiya A, Okabe M, Kawarai M et al. (2012) Development of an all-in-one inducible lentiviral vector for gene specific analysis of reprogramming. PLOS ONE 7: e41007. doi:10.1371/journal.pone.0041007. PubMed: 22815895. 22815895 PMC3399796

[36] Marchion DC, Bicaku E, Daud AI, Sullivan DM, Munster PN (2005) Valproic acid alters chromatin structure by regulation of chromatin modulation proteins. Cancer Res 65: 3815-3822. doi:10.1158/0008-5472.CAN-04-2478. PubMed: 15867379. 15867379

[37] Silva J, Nichols J, Theunissen TW, Guo G, van Oosten AL et al. (2009) Nanog is the gateway to the pluripotent ground state. Cell 138: 722-737. doi:10.1016/j.cell.2009.07.039. PubMed: 19703398. 19703398 PMC3437554

[38] Theunissen TW, van Oosten AL, Castelo-Branco G, Hall J, Smith A et al. (2011) Nanog overcomes reprogramming barriers and induces pluripotency in minimal conditions. Curr Biol 21: 65-71. doi:10.1016/j.cub.2010.11.074. PubMed: 21194951. 21194951 PMC3025321

[39] Gomyo Y, Sasaki J, Branch C, Roth JA, Mukhopadhyay T (2004) 5-aza-2'-deoxycytidine upregulates caspase-9 expression cooperating with p53-induced apoptosis in human lung cancer cells. Oncogene 23: 6779-6787. doi:10.1038/sj.onc.1207381. PubMed: 15273730. 15273730

[40] Yu J, Vodyanik MA, Smuga-Otto K, Antosiewicz-Bourget J, Frane JL et al. (2007) Induced pluripotent stem cell lines derived from human somatic cells. Science 318: 1917-1920. doi:10.1126/science.1151526. PubMed: 18029452. 18029452

[41] West JA, Viswanathan SR, Yabuuchi A, Cunniff K, Takeuchi A et al. (2009) A role for Lin28 in primordial germ-cell development and germ-cell malignancy. Nature 460: 909-913. PubMed: 19578360. 19578360 10.1038/nature08210 PMC2729657

[42] Polo JM, Anderssen E, Walsh RM, Schwarz BA, Nefzger CM et al. (2012) A molecular roadmap of reprogramming somatic cells into iPS cells. Cell 151: 1617-1632. doi:10.1016/j.cell.2012.11.039. PubMed: 23260147. 23260147 PMC3608203

[43] Mikkelsen TS, Hanna J, Zhang X, Ku M, Wernig M et al. (2008) Dissecting direct reprogramming through integrative genomic analysis. Nature 454: 49-55. doi:10.1038/nature07056. PubMed: 18509334. 18509334 PMC2754827

[44] Sridharan R, Tchieu J, Mason MJ, Yachechko R, Kuoy E et al. (2009) Role of the murine reprogramming factors in the induction of pluripotency. Cell 136: 364-377. doi:10.1016/j.cell.2009.01.001. PubMed: 19167336. 19167336 PMC3273494

[45] Buehr M, McLaren A, Bartley A, Darling S (1993) Proliferation and migration of primordial germ cells in We/We mouse embryos. Dev Dyn 198: 182-189. doi:10.1002/aja.1001980304. PubMed: 8136523. 8136523

[46] Chan EM, Ratanasirintrawoot S, Park IH, Manos PD, Loh YH et al. (2009) Live cell imaging distinguishes bona fide human iPS cells from partially reprogrammed cells. Nat Biotechnol 27: 1033-1037. doi:10.1038/nbt.1580. PubMed: 19826408. 19826408

[47] Plath K, Lowry WE (2011) Progress in understanding reprogramming to the induced pluripotent state. Nat Rev Genet 12: 253-265. doi:10.1038/nrg2955. PubMed: 21415849. 21415849 PMC3273493

[48] Chambers I, Silva J, Colby D, Nichols J, Nijmeijer B et al. (2007) Nanog safeguards pluripotency and mediates germline development. Nature 450: 1230-1234. doi:10.1038/nature06403. PubMed: 18097409. 18097409

