# Supplementary material for: Generation of Human Induced Pluripotent Stem Cells Using Epigenetic Regulators Reveals a Germ Cell-Like Identity in Partially Reprogrammed Colonies
Source: PLoS One. 2013 Dec 12;8(12):e82838. doi: 10.1371/journal.pone.0082838 (PMC3861446; doi:10.1371/journal.pone.0082838)
Supplement: Figure S1 — Analysis of DNMT family member expression in additional hESC and hiPSC lines. The expression of each member of the DNA methyltransferase (DNMT) family was analyzed in undifferentiated (D0 for Day 0) as well as Day 7 (D7), Day 14 (D14) and Day 21 (D21) differentiated human embryonic stem cells (hESCs) lines, HSF8 and HSF10, the original adult dermal fibroblasts (HUF5), undifferentiated (D0) human induced pluripotent stem cells (hiPSCs; clone 3), D7 and D14 differentiated hiPSCs by microfluidic Quantitative-PCR (Q-PCR). (DOCX) [file pone.0082838.s001.docx]

**
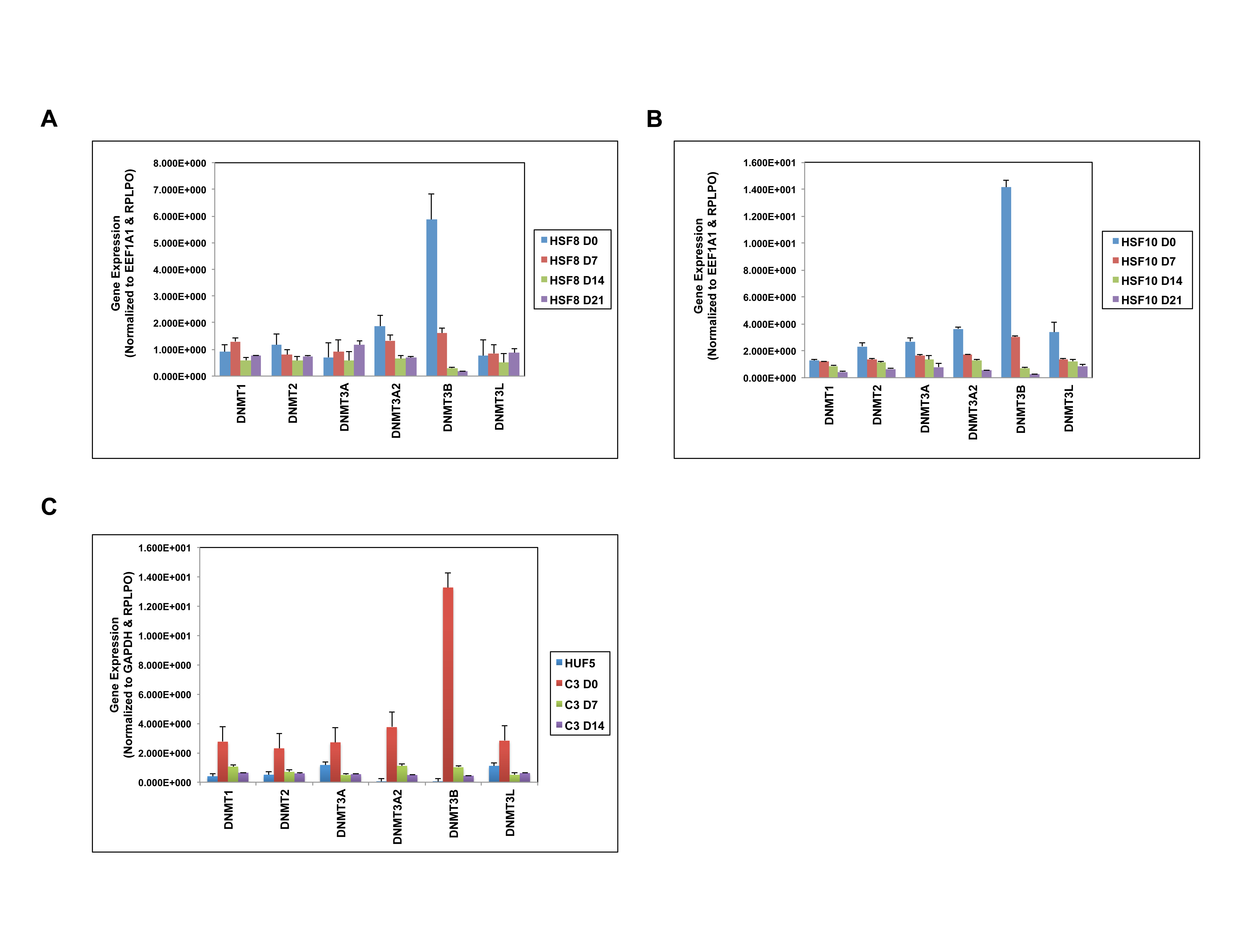
**

**Supplementary Figure 1. Analysis of DNMT family member expression in additional hESC and hiPSC lines.** The expression of each member of the DNA methyltransferase (DNMT) family was analyzed in undifferentiated (D0 for Day 0) as well as Day 7 (D7), Day 14 (D14) and Day 21 (D21) differentiated human embryonic stem cells (hESCs) lines, **(A)** HSF8 and **(B)** HSF10, by microfluidic Quantitative-PCR (Q-PCR). **(C)** Similar Q-PCR analysis of DNMT expression in the original adult dermal fibroblasts (HUF5), undifferentiated (D0) human induced pluripotent stem cells (hiPSCs; clone #3), D7 and D14 differentiated hiPSCs. Cycle threshold (Ct) values were normalized to the two most stable housekeeping genes and graphed as shown. Note that the expression of DNMT3B is elevated in undifferentiated hESCs and hiPSCs, but not HUF5 fibroblasts, and its expression decreases with differentiation.
