# Supplementary material for: Generation of Human Induced Pluripotent Stem Cells Using Epigenetic Regulators Reveals a Germ Cell-Like Identity in Partially Reprogrammed Colonies
Source: PLoS One. 2013 Dec 12;8(12):e82838. doi: 10.1371/journal.pone.0082838 (PMC3861446; doi:10.1371/journal.pone.0082838)
Supplement: Figure S2 — Quantification of histone-modifying enzyme expression in other hESCs and hiPSC lines. The expression of members from each class of histone-modifying enzymes was analyzed in undifferentiated (D0 for Day 0) as well as Day 7 (D7), Day 14 (D14) and/or Day 21 (D21) differentiated human embryonic stem cells (hESCs), HSF8 and HSF10, the original adult dermal fibroblasts (HUF5), undifferentiated (D0) human induced pluripotent stem cells (hiPSCs; clone 2), D7 and D14 differentiated hiPSCs by microfluidic Quantitative-PCR (Q-PCR). (DOCX) [file pone.0082838.s002.docx]

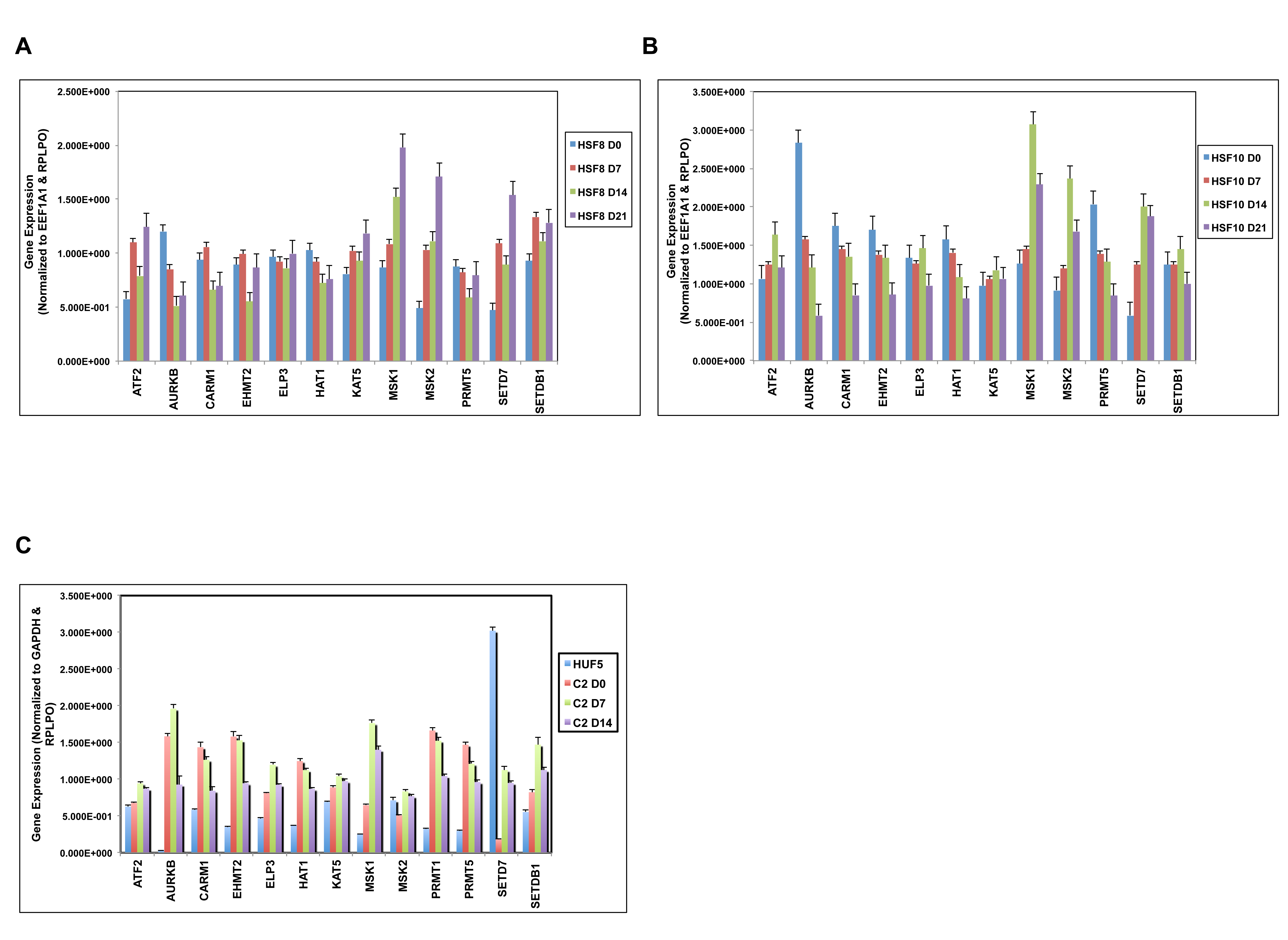


**Supplementary Figure 2. Quantification of histone-modifying enzyme expression in other hESCs and hiPSC lines.** The expression of members from each class of histone-modifying enzymes was analyzed in undifferentiated (D0 for Day 0) as well as Day 7 (D7), Day 14 (D14) and/or Day 21 (D21) differentiated human embryonic stem cells (hESCs), **(A)** HSF8 and **(B)** HSF10, by microfluidic Quantitative-PCR (Q-PCR). **(C)** Similar Q-PCR analysis of histone-modifying enzyme expression in the original adult dermal fibroblasts (HUF5), undifferentiated (D0) human induced pluripotent stem cells (hiPSCs; clone #2), D7 and D14 differentiated hiPSCs. Cycle threshold (Ct) values were normalized to the two most stable housekeeping genes and graphed as shown. Note the high levels of AURKB and PRMT5 expression in undifferentiated hESCs and hiPSCs and the elevated expression of SETD7 in HUF-5 fibroblasts, which decreases with reprogramming and increases with differentiation.
