# Supplementary material for: Generation of Human Induced Pluripotent Stem Cells Using Epigenetic Regulators Reveals a Germ Cell-Like Identity in Partially Reprogrammed Colonies
Source: PLoS One. 2013 Dec 12;8(12):e82838. doi: 10.1371/journal.pone.0082838 (PMC3861446; doi:10.1371/journal.pone.0082838)
Supplement: Figure S3 — Brightfield and fluorescent imaging of additional colonies obtained from human adult fibroblasts. Human adult dermal fibroblasts (HUF1 or HUF5) were nucleofected with DNMT3B-GFP and SETD7-MO or DNMT3B-GFP, SETD7-MO, AURKB and PRMT5 and colony formation assessed via brightfield and fluorescent imaging. (DOCX) [file pone.0082838.s003.docx]

**
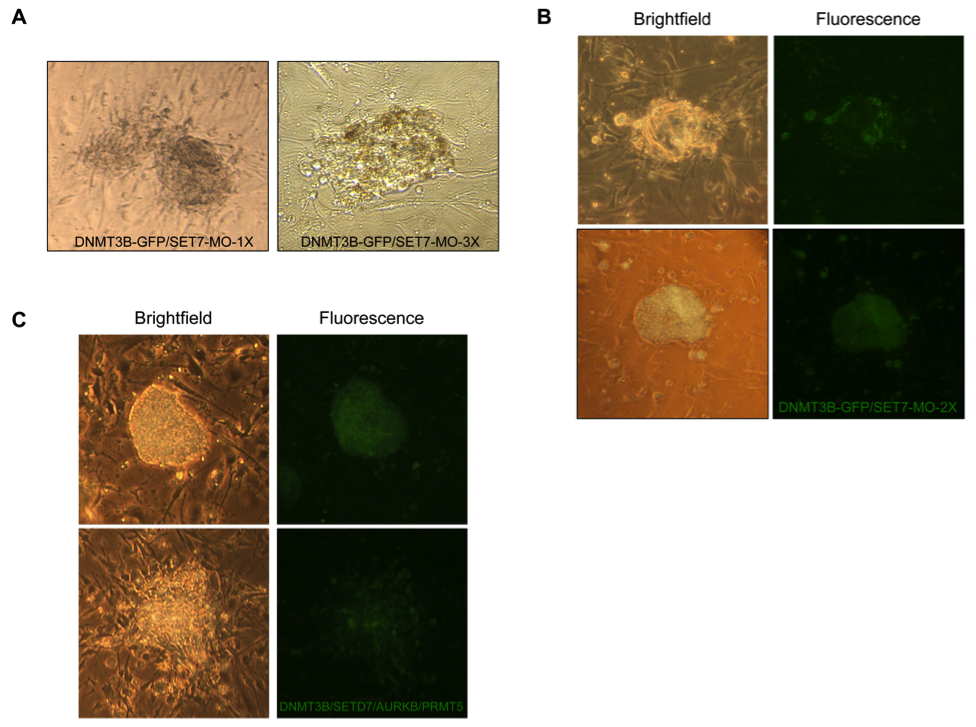
**

**Supplementary Figure 3. Brightfield and fluorescent imaging of additional colonies obtained from human adult fibroblasts.** Human adult dermal fibroblasts were nucleofected with DNMT3B-GFP and SETD7-MO and colony formation assessed via brightfield and fluorescent imaging. **(A)** The formation of several small clones was observed from HUF5 cells transfected with DNMT3B-GFP and SETD7-MO one time (1X; left), which proliferated until day 10 and then underwent senescence after approximately 14 days post-nucleofection. Transfecting HUF5 fibroblasts three times (3X; right) resulted in cell death due to cellular detachment for nucleofection even in the presence of conditioned media on matrigel or mouse embryonic fibroblasts (MEFs). **(B)** The transfection of HUF5 cells two times (2X) on Day 1 and Day 3 was the most efficient in terms of reprogramming efficiency and number of clones obtained. **(C)** The induction of AURKB and PRMT5 expression together with DNMT3B over expression and SETD7 silencing resulted in cell death and reduced colony formation, which may be due to the large amount of DNA needed to transfect the HUF1 fibroblasts with all four factors.
