# Supplementary material for: Generation of Human Induced Pluripotent Stem Cells Using Epigenetic Regulators Reveals a Germ Cell-Like Identity in Partially Reprogrammed Colonies
Source: PLoS One. 2013 Dec 12;8(12):e82838. doi: 10.1371/journal.pone.0082838 (PMC3861446; doi:10.1371/journal.pone.0082838)
Supplement: Figure S4 — Additional colonies obtained from various reprogramming strategies using human neonatal fibroblasts. Neonatal human foreskin fibroblasts (HFF-1) were treated with 5-Aza-2´-deoxycytidine (AZA) and/or Valproic Acid (VPA) in combination with DNMT3B-GFP, SETD7-MO and NANOG or DNMT3B-GFP, SETD7-MO, NANOG, SV40 and hTERT nucleofection and colony formation assessed via brightfield imaging. (DOCX) [file pone.0082838.s004.docx]

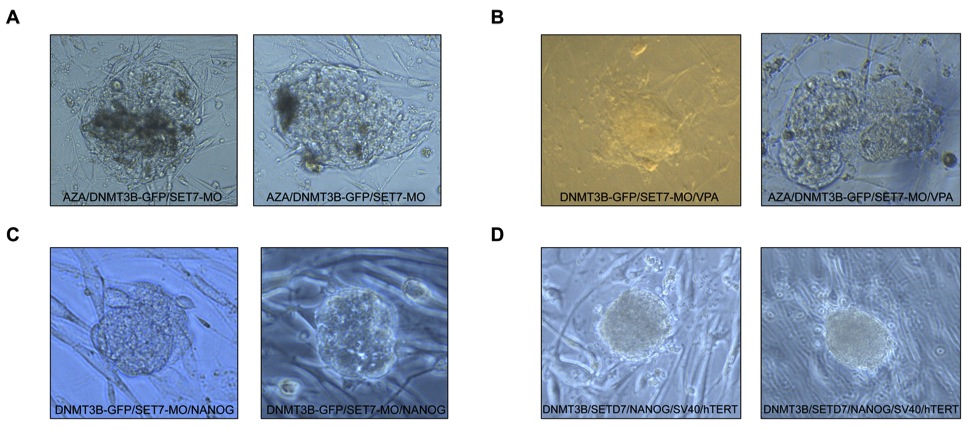


**Supplementary Figure 4. Additional colonies obtained from various reprogramming strategies using human neonatal fibroblasts. (A)** Neonatal human foreskin fibroblasts (HFF-1) were pre-incubated with 5-Aza-2´-deoxycytidine **(**AZA) for three days and then nucleofected with DNMT3B-GFP and SETD7-MO, which resulted in decreased colony formation and AZA-induced cell death. **(B)** The addition of Valproic Acid (VPA) to the HFF-1 culture media with or without AZA reduced cell death and significantly increased colony formation. **(C)** Decreased colony formation was also observed in HFF-1 cells transfected DNMT3B-GFP, SETD7-MO and NANOG as well as with **(D)** DNMT3B-GFP, SETD7-MO, NANOG, SV40 and hTERT, which is likely the result of the large amount of DNA needed to nucleofect the neonatal fibroblasts with more than two factors. However, the addition of NANOG to the reprogramming cocktail changed the morphology of the colonies to a more embryonic germ (EG) cell-like appearance.
