# Supplementary material for: Generation of Human Induced Pluripotent Stem Cells Using Epigenetic Regulators Reveals a Germ Cell-Like Identity in Partially Reprogrammed Colonies
Source: PLoS One. 2013 Dec 12;8(12):e82838. doi: 10.1371/journal.pone.0082838 (PMC3861446; doi:10.1371/journal.pone.0082838)
Supplement: Table S1 — The reprogramming efficiency of each transfection approach. A table displaying the cell type, transfection method, reprogramming factors and treatment conditions used for each transfection approach in this study. (DOCX) [file pone.0082838.s005.docx]

| **Cell type, transfection method, reprogramming factors and treatment conditions** | **Number of clones per 1 million cells transfected** | **Reprograming efficiency** |
| --- | --- | --- |
| AHDF nucleofected with DNMT3B + SETD7-MO | 30 | 0.003% |
| HFF-1 nucleofected with DNMT3B + SETD7-MO + AURKB + PRMT5 | 15 | 0.0015% |
| HFF-1 nucleofected with  DNMT3B + SETD7-MO + NANOG + SV40 + hTERT | 12 | 0.0012% |
| HFF-1 pre-treated with AZA and nucleofected with DNMT3B + SETD7-MO for culture in media containing VPA | 35 | 0.0035% |
| HFF-1 pre-treated with AZA and nucleofected with DNMT3B + SETD7-MO | 18 | 0.0018% |
| HFF-1 nucleofected with SETD7-MO and then transfected with DNMT3B + SV40 + hTERT using cationic lipid reagent | 50 | 0.005% |

**Supplementary Table 1.** The reprogramming efficiency of each transfection approach. A table displaying the cell type, transfection method, reprogramming factors and treatment conditions used for each transfection approach in this study. The reprogramming efficiency was calculated by dividing the number of clones obtained by the number of cells transfected, which was approximately 1 million per treatment group. Note that the highest reprogramming efficiencies were observed with DNMT3B and SETD7-MO transfection in the absence or presence of SV40, hTERT and VPA. However, similar to pre-treatment with AZA, the addition of SV40 and hTERT to the reprogramming factor cocktail induced cell death in colonies following transfection.
