# Supplementary material for: Generation of Human Induced Pluripotent Stem Cells Using Epigenetic Regulators Reveals a Germ Cell-Like Identity in Partially Reprogrammed Colonies
Source: PLoS One. 2013 Dec 12;8(12):e82838. doi: 10.1371/journal.pone.0082838 (PMC3861446; doi:10.1371/journal.pone.0082838)
Supplement: Table S2 — Comparison of gene expression ratios between cell types. A table comparing global gene expression levels of pluripotency factors, the candidate reprogramming factors (DNMT3B and/or SETD7) and germ cell markers in the original HUF5 adult dermal human fibroblasts, following transfection with DNMT3B and/or SETD7-MO with passage (P) and clone (C) numbers, and conventionally generated induced pluripotent stem cells (iPS) on Day 0, 7 and 14 of differentiation with and without Bone Morphogenetic Proteins (BMPs). (DOCX) [file pone.0082838.s006.docx]

| **Cell type with transfection approach** | **OCT4** | **SOX2** | **NANOG** | **TERT** | **DNMT3B** | **SETD7** | **DAZL** | **IFITM1** | **PRDM14** | **PUM1** | **PUM2** | **STELLAR** | **VASA** | **SCP3** | **STRA8** |
| --- | --- | --- | --- | --- | --- | --- | --- | --- | --- | --- | --- | --- | --- | --- | --- |
| HUF1 DNMT3B P2 | +++ | - | - | - | +++++ | - | - | - | ++ | 1.5 | + | - | - | - | - |
| HUF5 DNMT3B P1 C1 | - | - | - | - | - | + | - | - | - | - | - | - | - | - | - |
| HUF5 DNMT3B P1 C2 | ++.5 | ++ | - | - | 0.25 | - | - | - | - | +++ | + | ++++ | - | - | - |
| HUF5 DNMT3B P1 C3 | - | - | - | - | - | + | - | 0.5 | - | - | - | - | - | - | - |
| HUF5 DNMT3B P2 | ++ | - | - | - | - | ++ | - | - | - | + | + | - | - | - | - |
| HUF1 DNMT3B + SETD7 P1 | +++ | + | - | +++ | ++ | - | - | - | - | - | - | - | - | - | - |
| HUF1 DNMT3B + SETD7 P2 | - | - | - | - | - | 1.5 | - | - | - | - | - | - | - | - | - |
| HUF5 DNMT3B + SETD7 P1 C1 | - | +++ | - | ++ | - | + | - | - | - | - | - | - | - | - | 0.5 |
| HUF5 DNMT3B + SETD7 P1 C2 | ++ | + | - | 0.5 | 0.5 | - | - | + | - | - | - | - | - | - | - |
| HUF5 | - | ++ | - | + | - | +++ | - | + | - | - | - | 0.25 | - | - | 0.5 |
| iPS-HUF5  -DAY 0 | +++ | ++ | +++ | 0.5 | 0.5 | 0.25 | ++ | - | - | - | - | 0.25 | 0.5 | + | 0.5 |
| iPS-HUF5  -DAY 7 | ++ | - | + | - | - | + | - | - | - | - | - | - | - | - | - |
| iPS-HUF5-DAY14 | + | - | 0.5 | - | - | + | - | - | - | - | - | - | - | - | - |
| iPS-HUF5-DAY7 BMPs | + | +.5 | + | +.5 | 0.5 | - | - | - | - | 0.5 | + | + | + | 0.5 | - |
| iPS-HUF5-DAY14 BMPs | 0.5 | 0.5 | - | + | - | +.5 | - | 0.5 | - | 0.5 | + | + | +.5 | + | - |

**Supplementary Table 2.** Comparison of gene expression ratios between cell types. A table comparing global gene expression levels of pluripotency factors, the candidate reprogramming factors (DNMT3B and/or SETD7) and germ cell markers in the original HUF5 adult dermal human fibroblasts, following transfection with DNMT3B and/or SETD7-MO with passage (P) and clone (C) numbers, and conventionally generated induced pluripotent stem cells (iPS) on Day 0, 7 and 14 of differentiation with and without Bone Morphogenetic Proteins (BMPs). Note the high levels of certain pluripotency and germ cell-specific genes in some of the transfected clones in comparison to the HUF5 fibroblasts and iPS cells. The differentiation of iPS cells in the presence of BMPs induces the expression of mid to late germ cell markers to suggest that the partially reprogrammed colonies exhibit characteristics of early germ cell-like cells. This table is an accompaniment to **Figure 5E**.
